# Supplementary material for: Kullback–Leibler Divergence of Sleep-Wake Patterns Related with Depressive Severity in Patients with Epilepsy
Source: Brain Sci. 2023 May 19;13(5):823. doi: 10.3390/brainsci13050823 (PMC10216093; doi:10.3390/brainsci13050823)
Supplement: Supplementary file 1 [file brainsci-13-00823-s001.zip › brainsci-2381610-supplementary.pdf]

Supplement

Supplement Table S1. Correlative analysis between HAMD-17 scores and external variables.

External variable	r-value	p-value
Sex	-0.12	0.35
Age	0.02	0.86
Age at epilepsy diagnosis	0.06	0.64
Epilepsy duration	-0.13	0.33
Epilepsy type	0.21	0.09
ASD numbers	0.01	0.98
MMSE	-0.27	0.04*
DS-forward	-0.10	0.45
DS-backward	-0.24	0.07
VFT-animals	-0.10	0.48
SDMT	-0.13	0.42
AVLT immediate recall	-0.18	0.19
AVLT delayed recall	-0.10	0.50
AVLT recognition	-0.05	0.74

Note: HAMD-17 = 17-item Hamilton Depression Rating Scale, MMSE = Mini-Mental State Examination, AVLT = Rey Auditory Verbal Learning Test, DS = digital span, SDMT = Symbol Digit Modalities Test, and VFT = verbal fluency test.

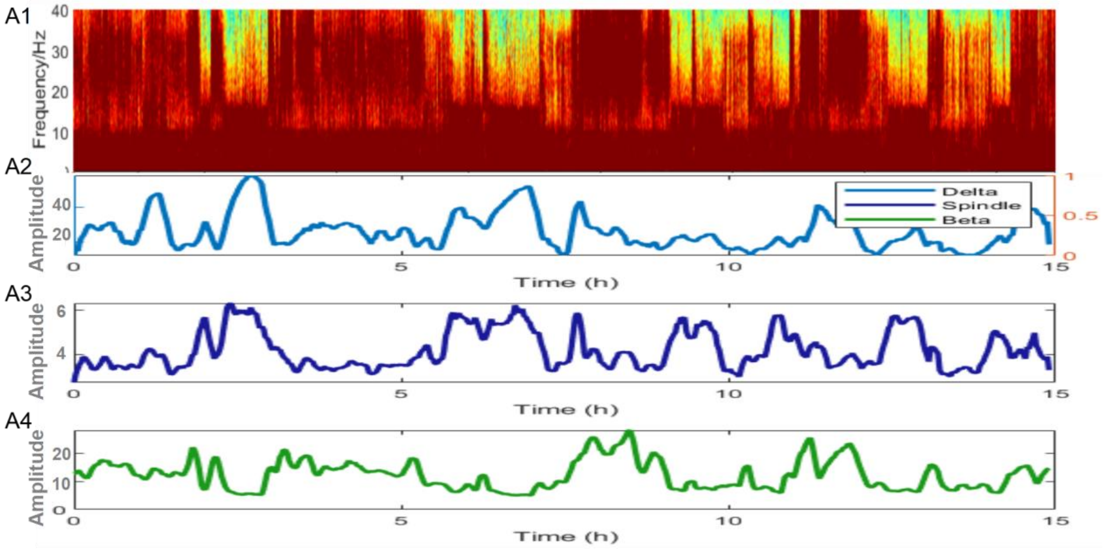

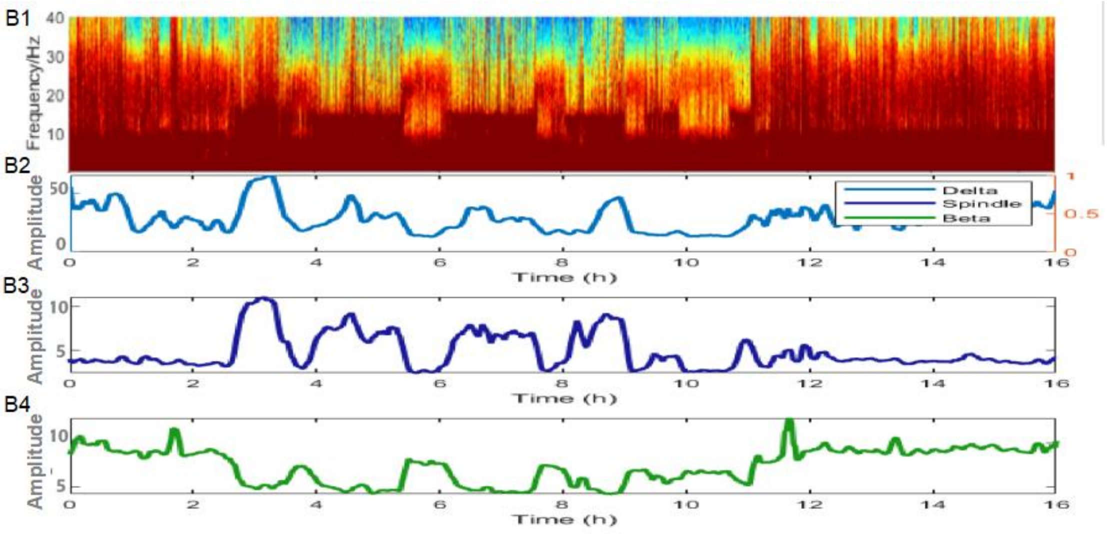

Supplement Figure S1. Spectrogram and envelope diagrams in patients with depression and without depression. (A) Patients with depression. A1: time-frequency diagram, A2: time-dependent envelope of delta bands, A3: time-dependent envelope of spindle bands A4:time-dependent envelope of beta bands; (B) Patients without depression. B1: time-frequency diagram, B2: time-dependent envelope of delta bands, B3: time-dependent envelope of spindle bands, B4:time-dependent envelope of beta bands.
